# The use of medical cannabis in pediatric palliative care: a case series

**DOI:** 10.1186/s13052-021-01179-1

**Published:** 2021-11-21

**Authors:** Antuan Divisic, Irene Avagnina, Valentina De Tommasi, Anna Santini, Laura Brogelli, Luca Giacomelli, Franca Benini

**Affiliations:** 1 Pediatric Pain and Palliative Care Service, Department of Women’s and Children’s Health, University Hospital, Padova, Italy; 2 Polistudium Srl, Milan, Italy

**Keywords:** cannabis, D9-tetrahydrocannabinol, Cannabidiol, Pediatric palliative care

## Abstract

**Background:**

Medical cannabis may be a useful tool for managing treatment-resistant epilepsy and chronic pain, which affect many patients in pediatric palliative care (PPC); however, little evidence is available in this setting.

**Case presentation:**

We aimed to describe a clinical experience in a setting where high-level evidence may not be obtained. We report our clinical experience in a pediatric palliative care department in Italy. Caregivers reported changes in intensity and frequency of pain and epilepsy events. Six patients received a titrated plant extract of *cannabis sativa* for 1 year. Only mild and transient adverse events occurred: drowsiness, euphoria, restlessness and tachycardia; the resolution was either spontaneous or obtained by modifying the administration schedule. Treatment was never discontinued. No overdoses occurred. All patients experienced seizures during the pre-treatment observation period, and obtained a reduction in seizure frequency, although with variable extent while receiving cannabis. In addition, a benefit on pain was observed, based on the caregiver’s evaluation, and a reduction of analgesic use.

**Conclusion:**

Our experience suggests that a titrated plant extract preparation of medical cannabis may be useful to control treatment-resistant pain and epilepsy in PPC patients.

## Background

Pediatric palliative care (PPC) aims to improve the quality of life of children with complex needs who are affected by a wide range of serious medical conditions for which no curative treatments are available. In this setting, a special focus on the care of families is pivotal. The role of the endocannabinoid system in epileptogenesis and pain inhibition has recently attracted attention in adult palliative care, based on the pharmacological basis to the use of exogenous cannabinoids to treat some of the more frequent and troublesome conditions of patients in palliative care, such as pain, poor appetite, weight loss, anxiety and treatment-resistant epilepsy [1, 2]. In addition, the use of medical cannabis in children is rapidly growing in various types of refractory epilepsy. However, the possible risk of cannabis use during childhood and adolescence is debated [3].

A systematic review of clinical trials assessing pharmaceutical-grade cannabidiol (CBD) in children with epilepsy, including data from 17 observational studies, showed that 20 mg/kg/day CBD was more effective than placebo in terms of seizure number reduction [4]. A meta-analysis including four clinical trials on CBD as adjunctive treatment in children with treatment-resistant Lennox–Gastaut and Dravet syndromes, found that seizure frequency was lower by 19.9% with 20 mg/kg/day CBD in comparison with placebo [5]. It has been suggested that various cannabinoids present in herbal extracts may interact synergistically to produce a greater effect compared with single compounds [6–8]. A higher responder rate was obtained with a cannabis extract that contained low levels of D9-tetrahydrocannabinol (THC) in comparison with studies using pharmaceutical-grade CBD [9]. Pamplona et al. obtained a reduction in seizure frequency, with lower doses of CBD, in a larger percentage of children taking herbal extract than in those receiving pharmaceutical-grade CBD (71% vs 46%; *p* ≤ 0.0001) [10].

Although conflicting data have been published on neuropathic pain, a systematic review in palliative medicine found limited evidence for the benefit of THC/CBD spray in the treatment of neuropathic pain, and a meta-analysis of 18 clinical studies on chronic pain and 776 patients found a moderate efficacy of treatment with cannabis [11–16].

Medical cannabis could be a useful tool for managing treatment-resistant epilepsy and chronic pain, which affect many patients in the PPC [17, 18]; however, evidence is available in this setting, where it seems impossible to carry out randomized trials or large observational studies. Even low-grade evidence is necessary to promote therapeutic options, which could improve the quality of life of very difficult patients. An attentive description of the case series may provide useful information. We report here our clinical experience with six patients in PPC who received a titrated plant extract of *cannabis sativa* for 1 year.

## Case presentation

In accordance with regional recommendations for the prescription of cannabinoids, medical cannabis treatment was proposed to six patients followed-up in the Centro Regionale Veneto di Cure Palliative Pediatriche e Terapia del Dolore of Padova Hospital, Italy – a referral center for PPC in northern Italy – who were suffering from treatment-resistant epilepsy and/or chronic pain and/or spasticity resistant to standard treatment lines, without contraindication to the use of medical cannabis due to psychiatric or cardiovascular disease. Data presented here cover an observation period of 1 month before initiation of cannabis and 12 months during treatment (except for patient 3). The previously prescribed antiepileptic and analgesic therapy were continued in concomitance with cannabis.

Treatment with FM2, with clinical outcomes, are reported in Table 1, symptom improvement is reported in Table 2, and the whole course is summarized in Table 3.

**Table 1:** Course of treatment with FM2, with clinical outcomes

Patient	Starting/maximal dose (mg/kg/day)	Effective dose (mg/kg/day)	Dosage changes (n)	Titration changes (n)	Daily frequency of seizures (beginning/best result; features)	% of days free from seizures (beginning/best result)	% of days free from pain (beginning/best result)	Adverse events with dose of FM2 at occurrence
1	0.11/059	0.55	7	10	0.11/0.26; tonic–clonic	89/89	100/100	No
2	0.11/0.52	0.38	5	9	8/2.31	0/0	3/82.8	Drowsiness with 0.52 mg/kg/day
3	0.1/0.48	0.48	4	3	3.57/2	0 / 33	28/100	Euphoria with 0.1 mg/kg/day
4	0.1/0.22	0.22	4	4	0.79/1	57/73	NA	Drowsiness: resolution with schedule change from 0.4 ml × 3 to 0.3 + 0.4 + 0.8 ml each day
5	0.12	0.2	4	2	5/3; absence and tonic–clonic	0/NA	25/58	Drowsiness: remission with schedule change from 0.65 ml three-times/day to 0.5 + 0.5 + 0.9 ml/day
6	0.71/1.2	0.44	3	6	0.23/0.14	NA	NA	Irritability with 1.2 mg/kg/day: dose reduction to 0.85 mg/kg/day Euphoria with 0.6 mg/kg/day: spontaneous resolution

**Table 2:** Symptom improvement following treatment with FM2, as evaluated by caregivers

Patient	Seizures	Pain	Spasticity	Restlessness	Sleep disorder	Reduced relationship
1	+/−	+/−	–	NA	–	+
2	+	+	–	+	+	–
3	+	+	+	+	+	+
4	+/−	–	+	–	+	+
5	+	+	–	+	–	+
6	+	+	–	–	+	+

+: Improvement of symptoms; +/−: Mild improvement of symptoms; −: No improvement of symptoms; NA: Not available

**Table 3:** Course of treatment with FM2, with clinical outcomes

	Dose (mg/kg/day)	Titration (mg/ml)	Duration (days)	Seizure n (daily frequency); features	Days free from seizures	Pain	Days free from pain (%)	Adverse events
**Patient 1**								
Starting dose	0.11	THC 5.2%; CDB 6.2%	9	1 (0.11) Tonic-clonic, no O_2_ desaturation	8/9	No	9 (100)	No
Change 1	0.21	Continued	15	9 (0.6); tonic-clonic, with O_2_ desaturation in 8/9	12	No	15 (100)	No
Change 2	0.41	Continued	9	3 (0.33); with O_2_ desaturation	7	No	9 (100)	
Change 3	0.52	THC 5.2%; CBD 12.5%	70	42 (0.6); O_2_ desaturation in 37/42		Yes	67 (96); analgesiscs 5-tims	No
Change 4	0.59	THC 5.2%; CDB 8%	24	9 (0.38); with O_2_ desaturation		Yes	21 (87.5)	
Change 5	0.55	THC 5.1%; CDB 8.3%	69	36 (0.52); O_2_ desaturation in 34/36		Yes	65 (94.2)	
Change 6	continued	THC 5.2%; CDB 8.4%	36	18 (0.5); O_2_ desaturation in 18/18		no	36 (100)	
Change 7	continued	THC 4.7%; CDB 7.6%	43	11 (0.26)		Yes	37 (86)	
Change 8	0.42	THC 4.5%; CDB 7.4%	37	12 (0.32)		No	37 (100)	
Change 9	0.56	THC 4.5%; CDB 7.3%	29	10 (0.34)		No	29 (100)	
Change 10	0.57	THC 4.6%; CDB 7.4%	19	13 (0.68)	17	No	19 (100)	
Change 11	0.55	THC 4.4%; CDB 7.1%	22	10 (0.45)	19	No	22 (100)	
**Patient 2**								
Starting dose	0.11	THC 6.5%; CDB 8.5%	33	266 (8)	0	Yes, moderate	1 (3)	
Change 1	0.2	Continued	33	191 (5.79)	0	Moderate	1 (3)	
Change 2	0.31	Continued	30	125 (4.17)	0	Mild	3 (10)	
Change 3	0.34	THC 5.8%; CDB 7.2%	26	157 (6.04)	0	Moderate	0	
Change 4	0.23	THC 2.7%; CDB 9.3%	31	100 (3.23)	0	Mild	2 (6.5)	
Change 5	0.21	THC 1.2%; CDB 1.6%	14	43 (3.07)	0	Moderate	0	
Change 6	0.51	THC 5.9%; CDB 8.3%	30	80 (2.66)	0	Mild	14 (48.3)	
Change 7	0.52	THC 6.1%; CDB 8.2%	38	116 (3.05)	0	Mild	20 (52.6)	Drowsiness
Change 8	0.34	THC 4%; CDB 6.2%	31	83 (2.68)	0	Mild	16 (51.6)	
Change 9	0.39	THC 4.6%; CDB 6.5%	29	73 (2.52)	0	Mild	18 (62)	
Change 10	0.4	THC 4.9%; CDB 6.6%	27	68 (2.52)	0	Very mild	18 (66.7)	
Change 11	0.38	THC 4.6%; CDB 6.3%	35	81 (2.31)		Very mild	20 (82.8)	
**Patient 3**								
Starting dose	0.1	na	50	150 (3.57); 50 with O_2_ desaturation	0	NRS = 1	14 (28)	Euphoria
Change 1	0.2		30	90 (3); tonic-clonic, 20 with O_2_ desaturation	0	NRS = 1	20 (67)	Euphoria
Change 2	0.31	THC 4.1%; CBD 2.9%	42	84 (2); with O_2_ desaturation	12 (29)	No	42 (100)	
Change 3	0.28	THC 3.7%; CBD 3.1%	60	120 (2); 22 with O_2_ desaturation	20 (33)	No	60 (100)	
Change 4	0.48	THC 5.4%; CBD 3.5%	90	374 (2)	60 (32.1)	No	90 (100)	
**Patient 4**								
Starting dose	0.1	THC 5%; CBD 6.7%	14	11 (0.79); no O_2_ desaturation	8 (57)	NRS = 4	NA	
Change 1	0.17	Continued	9	15 (1.67)	4 (44)	NA	NA	
Change 2	0.22	Continued	53	60 (1.13	33 (62)	NA	NA	Drowsiness Resolution with schedule change from 0.4 ml × 3 to 0.3 + 0.4 + 0.8 ml each day
Change 3	Continued (0.3 + 0.4 + 0.8 ml/day)	THC 4.7%; CBD 9.9%	60	79 (1.23)	38 (59.4)	NA	NA	
Change 4	0.1 (0.4 ml × 3 daily)	THC 2%; CBD 8.9%	84	85 (1)	49 (58.3)	NA	NA	
Change 5	0.14 (0.4 + 0.5 + 0.9 ml/day)	THC 2.3%; CBD 2.9%	27	34 (1.26)	14 (52)	NA	NA	
Change 6	0.22	THC 4.7%; CBD 5.5%	30	30 (1)	22 (73)	NA	NA	
Change 7	0.17	Continued	30	30 (1)	17 (58)	NA	NA	
Change 8	0.22	Continued	60	81 (1.37)	NA	NA	NA	
**Patient 5**								
Starting dose	0.12	THC 6%; CBD 8%	8	40 (5)	0	3	2 (25)	
Change 1	0.23	Continued	7	28 (4); absence and tonic-clonic		2	2 (29)	
Change 2	0.4	Continued	15	60 (4)		2	7 (47)	
Change 3	0.62	THC 6.4%; CBD 8.9%	26	78 (3)	0	2	15 (58)	Drowsiness: remission with schedule change from 0.65 ml three-times/day to 0.5 + 0.5 + 0.9 ml/day
Change 4	0.42	Continued	77	231 (3)		2	44 (57)	Euphoria
Change 5	0.38	THC 5.9%; CBD 10%	240	720 (3)		2		Euphoria: remission with dose reduction to 0.2, schedule 0.2 + 0.3 + 0.9 ml/day
**Patient 6**								
Starting dose	0.71	THC 6.74%	39	9 (0.23)		Analgesics 12-times		
Change 1	1.2	THC 11.5%; CBD 14.1%	27	6 (0.22)	NA	Analgesics 9-times		Irritability: dose reduction to 0.85 mg/kg/day
Change 2	0.4	THC 5.42%; CBD 7.87%	86	13 (0.15)		Analgesics 13-times	NA	
Change 3	0.6	THC 10.5%; CBD 9.1%	56	8 (0.14)	NA	Analgesics 13-times	NA	Euphoria: spontaneous resolution
Change 4	0.4	THC 4.8%; CBD 7.4%	38	6 (0.16)	NA	Analgesics 10-times	NA	
Change 5	0.44	THC 4.14%; CBD 5.61%	84	28 (0.33)	NA	Analgesics 64-times	20 (23)	Reduced appetite
Change 6	0.49	THC 4.6%; CBD 6.16%	28	NA	NA	No pain	0 (100)	Restlessness and tachycardia

### Medical cannabis

Patients were treated with cannabis FM2 (Farmaceutico Militare 2 cannabinoids), produced by Stabilimento Farmaceutico Militare, Florence, Italy [19]. FM2 is a powder of unfertilized female inflorescences containing 5–8% THC and 7.5–12% CBD. FM2 was prepared every month as galenic 10% extract in olive oil [20], and administered alone or in lipophilic beverages, either orally or enterally, three-times/day. Titration of THC and CBD was measured by liquid chromatography column mass spectrometry and recorded every month. The therapeutic dosage was referred to the THC content of the preparation. The minimal starting dose was 0.1 mg/kg/day and was increased after at least 7 days of 0.1 mg/kg/day, up to the minimal effective dose (maximal dosage was 1 mg/kg/day). According to the efficacy criteria of regional recommendations, the dose could be increased only if pain were not reduced by ≥20% or the number/duration of seizures was not reduced by ≥30%.

### Assessments

The following data were recorded: daily frequency of seizures (treatment success was defined as reduction ≥50% of seizures frequency) and use of rescue therapy [21–23]; pain intensity on an indirect numeric rating scale (NRS), frequency of pain flare and use of analgesics; changes in sleep, behavior, relationship, appetite and spasticity; the impact of treatment on the quality of life of the patient’s family; and adverse events. The patient’s main caregiver filled in the Italian version of the Pediatric Quality of Life Inventory TM (PEDsQL) questionnaire [24] at baseline and every 3 months up to 1 year of cannabis treatment, assisted by a psychologist (except patient 6). The questionnaire contains 5 subscales evaluating the caregiver (health and physical activity, emotional condition, social life, cognition, communication, and care), and two subscales evaluating the family (daily life activities and relationships). Answers were given on a 5-point Likert scale (high scores indicated a low impact and low scores indicated a serious impact).

### Ethics

Caregivers provided written consent. Cannabinoids were prescribed following regional recommendations for the prescription of cannabinoids. The procedures followed and described here were in accordance with the Declaration of Helsinki as revised in 2013.

### Patient #1

A 4-year-old boy with epileptic encephalopathy and mixed chronic pain had mild spasticity, sleep disorder, and severe neurological impairment. He was nourished by percutaneous endoscopic gastrostomy (PEG) and had a bodyweight of 13 kg. Uncontrolled epilepsy was the main complaint, and irritability episodes were frequent and interpreted as poor pain control. Data collection started 27 days before FM2 treatment, in a period without interventions on therapies and/or care. Before initiation of cannabis, the patient was free from pain on 23/27 (85%) days. In total, 15 tonic–clonic seizures occurred in 27 days (0.6/day), and 22/27 days (81.5%) were free from seizures. All seizures presented in a cluster, and 14/15 seizures led to oxygen desaturation. Seizures rescue therapy was used four-times.

FM2 treatment started with the dose of 0.11 mg/kg/day, which was increased weekly up to 0.59 mg/kg/day, without adverse events (Table 3).

Overall, fairly good control of pain was obtained, with a 15% increase of days free from pain. The proportion of days without seizures was 81.5% before the use of cannabis and was slightly reduced during the first month of treatment; it was then progressively increased, up to 90%. The intensity of seizure clusters was reported by the caregiver to be reduced. An overall reduction of irritability and an improvement of relationships were observed.

The PEDsQL questionnaire showed that the disease impact on the family was reduced after 1 year of cannabis use (total score was 47.92 at baseline and 59.72 at observation end). Improvement was obtained in the subscales emotional condition, care, daily life activities and family relationships.

### Patient #2

An 18-year-old boy, affected with lissencephaly, severe neurological impairment, nourished by PEG, with a bodyweight of 33 kg, and spasticity, had drug-resistant epilepsy on treatment with benzodiazepines, phenobarbital, valproate and topiramate. He also received gastroprotective drugs and food supplementation. He suffered from chronic mixed pain, resistant to treatment with non-steroid anti-inflammatory drugs (NSAIDs) + opioids + corticosteroids. He was observed for 13 days before starting cannabis treatment while continuing the previously prescribed therapy. The pain was reported each day, in this period, with a mean NRS scale intensity = 3.5. Overall, 113 seizures occurred, with a daily frequency = 8.7, and no days were free from episodes. Seizures presented as spasms in 41 cases and tonic–clonic episodes in 72 cases. Seizures never led to oxygen desaturation, and rescue therapy was not used.

FM2 dose was initially 0.11 mg/kg/day and increased weekly up to 0.52 mg/kg/day (Table 3). The patient tolerated the treatment with FM2, with only a transient episode of drowsiness. He obtained an improved control of pain (from 0 days without pain to 80% of days without pain), which was moderate before treatment, together with a relevant reduction of seizure frequency, from 8.7/day to 2.3/day. At the end of the observation, the impact of disease on the family quality of life was not changed, as the PEDsQL total score was 34 at baseline and 37 at 12 months after initiation of cannabis. A little improvement was observed in the subscales emotional condition, social life and cognition, while the impact on care and daily life activities was increased.

### Patient #3

A 19-year-old boy who had a kidney transplant was affected with coenzyme Q10 deficiency, nurtured by PEG, and had a bodyweight of 34 kg. Chronic mixed pain, severe neurological impairment, mild spasticity and agitation were present. He was treated with benzodiazepines, phenobarbital, oxcarbazepine, proton pump inhibitors, corticosteroids and immunosuppressors. On-demand analgesic treatment was based on NSAIDs + opioids + corticosteroids. During the pre-cannabis 30-day period of observation, he received analgesics 12-fold, and the mean pain intensity, as caregiver-evaluated NRS was 4; 12/30 (40%) days were free from pain. Seizures occurred every day with a frequency of 6 episodes/day (total number of seizures was 180); all seizures were tonic–clonic, and 120 episodes lasted > 5 min; rescue therapy was administered three-times.

FM2 was started with 0.1 mg/kg/day and the dose increased weekly up to 0.48 mg/kg/day. At the highest dose, the patient had mild euphoria, which spontaneously remitted (Table 3).

He obtained a relevant reduction of seizure frequency (2 seizures/day) and pain control (zero analgesic treatment) with the use of medical cannabis; in addition, a positive effect was observed on spasticity, agitation, sleep disorder and relationship. Clinical data are available for 6 months of observation because the caregiver compliance was poor.

The disease impact on the family quality of life was reduced after 1 year of treatment with medical cannabis. The total PEDsQL score was 18 at baseline and 38 at the observation end. Improvements were observed in the areas of health and physical activity, emotional condition, social life, cognition, communication, care and family relationships. Only the impact on daily life activities was unchanged.

### Patient #4

A 14-year-old girl was affected with Rett’s syndrome, had moderate cognitive dysfunction, and had no particular pain. She had a bodyweight of 35 kg, was nurtured by PEG, presented with mild spasticity and restlessness. Antiepileptic treatment was based on valproate and benzodiazepine when needed; she also received a proton pump inhibitor and food supplementation. During the 30 days of observation before cannabis initiation, she had 58 tonic–clonic seizures (1.9/day), 33 of which lasted > 5 min; 9 (30%) days were free from seizures. No pain was reported from caregivers, but daily episodes of restlessness were observed. FM2 treatment was initiated with the daily dosage of 0.1 mg/kg/day, and the dose increased up to 0.22 mg/kg/day (Table 3).

Drowsiness was observed with the maximal dose; this adverse event resolved after the dosage schedule was changed from 33% of total dose three-times/day to 20% + 30% + 50% of total dose respectively in the morning, afternoon and evening. Overall, the patient obtained a limited reduction of seizure frequency and restlessness, while spasticity, sleep and relationship ability were improved.

The disease impact on the family quality of life was little changed after 1 year of treatment; PEDsQL total score was 52 at baseline and 54.8 at observation end. While the social life subscale score was improved from 75 to 175, the emotional condition score deteriorated from 250 to 175.

### Patient #5

A 17-year-old boy, with a bodyweight of only 20 kg, affected with epileptic encephalopathy, severe psychomotor impairment, had mixed pain, spasticity, restlessness and sleep disorder. He was treated with benzodiazepines, valproate, topiramate and baclofen, with proton pump inhibitor and food supplementation. Analgesic therapy was based on NSAIDs, opioids and corticosteroids. He was observed for 30 days before initiation of cannabis. In this period, mean pain intensity at NRS scale was 7, analgesic treatment was used 16-times, and 8/30 (27%) days were free from pain. He had 300 (10/day) absence and tonic–clonic type seizures, all lasting < 5 min and without oxygen desaturation. No days free from seizures were observed.

FM2 treatment was started with 0.12 mg/kg/day, and increased weekly to 0.62 mg/kg/day. At the maximal dose, drowsiness occurred and remitted after the administration schedule was changed from 33% of total dose three-times/day to 25% + 25% + 50% of total dose in the morning, afternoon and evening, respectively. After dose reduction to 0.42 mg/kg/day, euphoria occurred and remitted after the dose was reduced to 0.2 mg/kg/day, of which 15% + 20% + 65% was administered in the morning, afternoon and evening, respectively (Table 3).

Overall, in addition to a relevant improvement of pain (mean NRS changed from 7 to 3, and 57% of days free from pain) and seizures (3 seizures/day), this patient obtained a reduction of restlessness and improvement of relationship ability.

The total PEDsQL score was 41 at baseline and was increased up to 56 at the observation end. The areas that improved after 1 year of treatment with cannabis were health and physical activity, emotional condition, care, daily life activities and family relationships. On the contrary, the impact on social life, cognition and communication was not improved.

### Patient #6

A 5-year-old female child, affected with epileptic encephalopathy, severe psychomotor impairment, moderate pain associated spasticity, with NRS as high as 10 (caregiver report). She also had restlessness and sleep disorder. Therapy was based on benzodiazepines, phenobarbital and oxcarbazepine. She received a proton pump inhibitor and food supplementation. The pain was treated with NSAIDs, opioids and baclofen.

Before FM2 treatment, she had 300 (10/day) absence and tonic–clonic type seizures, without oxygen desaturation. Pain occurrence and intensity were not recorded.

At the beginning of the observation period, she was treated with FM20.71 mg/kg/day, prescribed by another center. Involuntarily, the dosage was increased to 1.2 mg/kg/day due to high titration (11.5% THC, 14.1% CBD), for 1 month (Table 3). As irritability ensued, the dosage was progressively reduced to 0.44 mg/kg/day.

During treatment with FM2, she had some adverse events: irritability requiring dose reduction, euphoria, loss of appetite, restlessness and tachycardia, which remitted spontaneously.

Through the observation period, this patient obtained an improvement of sleep disorder and relationship ability; the caregiver reported a reduction of pain intensity (reduced use of analgesics) and seizure frequency (0.33/day). The impact of disease on family quality of life could not be evaluated. The PEDsQL questionnaire could not be completed because this patient had already received cannabis before our observation, and a baseline was unavailable.

## Discussion

We report the clinical course of six patients in the PPC setting, all presenting with resistant symptoms and “global suffering”, who had received cannabis therapy for treatment-resistant epilepsy and chronic pain for 1 year. The treatment with medical cannabis in our setting appears to be feasible and safe. A titrated plant extract was used, which was administered in an oily vehicle either orally or enterally. The extract was titrated every month and the dosage was calculated and tailored according to the relative THC content of the preparation. The effective dosage was reached by progressively increasing the daily dose [18]. This method allowed the use of a plant extract, with the benefit of the synergistic activity of several cannabinoids, and possibly of other plant components, together with a strict and consistent dosage of the drug. Using titrated preparations, dose adjustments could be accurate, and administrations could be reliably recorded. In addition, the oily extract of cannabis was easily storable and administrable by caregivers. Indeed, the six caregivers, when asked, answered that they did not want to discontinue cannabis. The assessment of results was based on the judgment of caregivers, and the most frequent symptoms were considered. This approach is in agreement with the objective of the clinical management of patients in the PPC. Our aim is to improve the quality of life, and we always rely on caregivers’ opinions for reports of treatment effects or adverse events.

In our experience, only mild and transient adverse events occurred: drowsiness, euphoria, restlessness and tachycardia; the resolution was either spontaneous or obtained by modifying the administration schedule. Treatment was never discontinued. No overdoses occurred, suggesting that the drug was safe and easily managed by caregivers. A safety concern in using medical cannabis in children is the risk of drug tolerance, which may be checked in palliative care patients by the caregivers and was not observed in our cases. In addition, sedation induced by cannabis may be an advantage for patients in PPC who have moderate/severe neurological impairment; so that higher doses may be tolerated in comparison with patients with better cognitive abilities, who view sedation as an adverse event. Similarly, the impact of medical cannabis on long-term neurological development, which is debated in the literature, is not an issue in patients with a life-limiting/threatening disease.

This report cannot be considered an observational study, and no statistical analysis of data can be proposed. Patients are heterogeneous in terms of main diagnosis, age, and pain and epilepsy severity at baseline. Nevertheless, we believe that some considerations may be drawn from this case series description and may be useful to clinicians dealing with PPC patients.

All patients experienced seizures during the observation period, and obtained a reduction in seizure frequency, although with variable extent; specifically, four out of six patients had a > 50% decrease in seizure frequency, which can be considered a threshold for efficacy [8]. The effect on chronic pain, in terms of use of analgesics, the intensity of episodes and days free from pain, was dissimilar in our patients, but caregivers reported an improvement of treatment-resistant pain. Pain intensity was expressed on the NRS scale based on the caregiver’s report; this may explain some inconsistency of our results. Improvement of pain was reported in the same setting by Doherty et al. [17], with different kinds of medical cannabinoids in 21 children, for any indication. These authors suggested that medical cannabis could be safely added to analgesic treatment in children with resistant pain.

Spasticity was improved only in two subjects. Kuhlen et al. [18] administered 2.5% oily THC solution and obtained the abolishment or marked improvement of severe refractory spasticity in 12/16 children or adolescents in palliative care.

## Conclusion

In conclusion, our experience showed that a titrated plant extract preparation of medical cannabis affected treatment-resistant pain and epilepsy in PPC patients in some cases; this therapy was effective in some patients and was not associated with adverse events which could discourage the use. Careful dosage control is necessary to meet the therapeutic interval; this can be obtained by a strict titration of herbal extract and relative dosage adjustments. Based on our experience, the efficacy and safety of cannabis in PPC patients should be further investigated by clinical studies: further studies statistically driven should demonstrate a significant effect, which at the moment is only descriptively observed.

## Data Availability

Data are available on request to the corresponding author.
